# Vehicle Driver Monitoring through the Statistical Process Control

**DOI:** 10.3390/s19143059

**Published:** 2019-07-11

**Authors:** Arthur N. Assuncao, Andre L. L. Aquino, Ricardo C. Câmara de M. Santos, Rodolfo L. M. Guimaraes, Ricardo A. R. Oliveira

**Affiliations:** 1 Instituto Federal de Educação, Ciência e Tecnologia do Sudeste de Minas Gerais, Santos Dumont, MG 36240-000, Brazil; 2 Departamento de Computação, Universidade Federal de Ouro Preto, Ouro Preto, MG 35400-000, Brazil; 3 Instituto de Computação, Universidade Federal de Alagoas, Maceió, AL 57072-970, Brazil

**Keywords:** driver monitor, lane departure, statistical process control

## Abstract

This paper proposes the use of the Statistical Process Control (SPC), more specifically, the Exponentially Weighted Moving Average method, for the monitoring of drivers using approaches based on the vehicle and the driver’s behavior. Based on the SPC, we propose a method for the lane departure detection; a method for detecting sudden driver movements; and a method combined with computer vision to detect driver fatigue. All methods consider information from sensors scattered by the vehicle. The results showed the efficiency of the methods in the identification and detection of unwanted driver actions, such as sudden movements, lane departure, and driver fatigue. Lane departure detection obtained results of up to 76.92% (without constant speed) and 84.16% (speed maintained at ≈60). Furthermore, sudden movements detection obtained results of up to 91.66% (steering wheel) and 94.44% (brake). The driver fatigue has been detected in up to 94.46% situations.

## 1. Introduction

There is a consensus regarding the high rate of traffic accidents caused by lane deviation due to momentary driver fatigue [1]. This high rate of road accidents, involving driver failures, such as tiredness and drowsiness, impose a significant burden on society, constituting a severe public health problem. Nowadays, the vehicle technologies mitigate the above problems by introducing into the vehicle several devices composed of both hardware for sensing and software for processing and decision making. Based on generated data, it is possible to provide different applications, on board or in a cellphone, capable of monitoring and interacting with both vehicle and driver behavior.

The Advanced Driver Assistance Systems (ADAS) [2] are those that exploit the knowledge of the environment based on advanced sensors. Galvani [3] presents the different levels of automated driving defined by Society of Automotive Engineers (SAE) (https://www.sae.org/): (i) *No automation*, the driver performs all driving tasks; (ii) *Driver Assistance*, the driver controls the vehicle, but the system includes some driving assist features in the vehicle design; (iii) *Partial automation*, the vehicle has combined automated functions like acceleration and steering, but the driver must remain engaged with the driving task and monitor the environment at all times; (iv) *Conditional automation*, the driver is a necessity but does not monitor the environment. The driver must be ready to control the vehicle at all times with notice; (v) *High automation*, the vehicle is capable of performing all driving functions under certain conditions. The driver has the option to control the vehicle; (vi) *Full automation*, the vehicle is capable of performing all driving functions under all conditions. The driver also has the option to control the vehicle. In particular, in this paper, we investigate the *driver assistance* level, i.e., the ADAS still leaves the authority to the driver, but we take care of specific driving functions based on conventional sensors.

Our study uses the vehicle motion information, coming from inertial sensors (accelerometer and gyroscope) in the steering wheel and brake pedal, and driver behavior based on the camera. The actions monitored are the lane department, sudden movements detection, and driver fatigue detection. The lane department is concerned with warning the driver when the vehicle begins to move out of its lane. The sudden movements detection is also to detect when the driver is getting drowsy but using sudden movements in the steering wheel and the brake pedal. Finally, the driver fatigue detection corresponds to detecting when the driver is getting drowsy through the eyes monitoring.

To perform this monitoring, we propose the use of the Statistical Process Control (SPC) [4]. In particular, we applied Exponentially Weighted Moving Average control graphs (EWMA) [5] from the SPC to identify the quality of safe driving. In an initial proposal, to lane departure detection, we use the EWMA in the analysis of the data from the *X* and *Y* axes of only one accelerometer and the combination of an accelerometer and a gyroscope positioned to the center of the steering wheel. After that, we evaluate the previous proposal considering sudden movements detection, by using the SPC applied to the accelerometer on the steering wheel and potentiometers on the pedals. Finally, for driver fatigue detection, we use a camera positioned in front of the driver, an analysis is performed based on the closed eyes percentage with the use of SPC, and the driver is alerted in case of fatigue.

In the evaluation, we considered different scenarios with several vehicles in a predefined route simulator, where the driver committed incorrect movements when the test evaluator requested. These movements do not differ from involuntary movements, because our method detects anomalies in the steering process, so any movement out of control may be detected. The experiments showed that the proposed methods and systems are viable alternatives to driver monitoring. In the initial experiment, we evaluate the lane departure detection with results of up to 76.92% (without constant speed) and 84.16% (speed maintained at ≈60). In the second experiment, we evaluate together sudden movements detection and driver fatigue, achieving, to sudden movements detection, results up to 91.66% (steering wheel) and 94.44% (brake); and, to driver fatigue, results up to 94.46% (blinks). The results reveal that we can apply our method to lane departure, fatigue, and sudden movement detection with acceptable accuracy. Among several contributions of this work, the introduction of SPC to driver monitoring is the main one. Additionally, the solution is low cost, allows the continuous monitoring, without human intervention, and is transparent to the driver. Besides that, the solution executes in a limited embedded system, thus must have a low time complexity cost. This restriction turns our solution more competitive against the traditional ones.

We organized the rest of the paper as follows: Section 2 shows the related works. Section 3 discusses the concepts related to Statistical Process Control and its EWMA graph. Section 4 shows the methods developed for monitoring drivers: Lane departure detection, driver fatigue detection, and sudden movements detection. Section 5 presents the evaluation of each method presented. Finally, Section 6 shows the conclusions and future work.

## 2. Related Work

Here we present works related to general ADAS systems [2,6], lane departure problem [7], driver fatigue, and sudden movements detections [8]. For ADASs, we analyze the number of solutions, the types of detection performed, and the issuance of alerts. For other ones, we analyze the work considering the approach used (computational vision, vehicle-based or physiological signals), the method cost, the use of computational resources, sensitivity to light, and other interferences.

The literature presents some well-established ADASs proposals, such as iCar [9] which is a system aimed at reducing accidents and assisting the driver in various aspects of driving, such as lane detection, pedestrian detection, car detection, driver fatigue detection and rearview assistance for parking. There is also the ADAS proposed by Wang et al. [10] which presents a system with self-learning, aiming to help the driver in the task of maintaining safety concerning the car-to-front, reducing their workload and reducing accidents and allowing control of cruise speed and frontal collision warning. Finally, the ADAS proposed by Chien et al. [11], driver assistance, and pedestrian safety system that detects lanes, vehicles, and pedestrians in front of the vehicle. These systems generally use a combination of various techniques, such as computational vision, feature extraction, machine learning, object recognition, and human-computer interaction.

For the lane departure problem, Sandström et al. [12] created a method with signals from the steering wheel, avoiding loss in weather conditions and bad roads. Satzoda et al. [13] provided a low-cost video-based system designed to assist the driver by issuing warnings during lane drifting and lane changes implemented on the Snapdragon^TM^ embedded computing processor setup with the camera on top of the windshield. Son et al. [14] show a system with a low-efficiency loss under the most varied lighting conditions. With the use of computer vision, it works even under adverse atmospheric conditions and at night. Thinking about low cost and low resource consumption, Jung et al. [15] developed a system with a camera located in the center of the vehicle facing the road and a computer with low processing power. Unlike our solution that uses a vehicle-based approach, these works use computer vision, which is the standard in the literature.

Sensitivity to light is a relevant factor in the lane departure detection and methods based on computer vision usually suffer from light interference. At this point, different works [12,16,17] do not suffer interference. Our proposal does not suffer interference, but it presents some disadvantages, like the dependence on the sensitivity of the steering wheel of the vehicle for the calibration, needing to calibrate the steering wheel a first time. Thus, it requires specific calibration for the coupled steering wheel. The financial and resource cost is also relevant. In this sense, in addition to our method, only the work of Jung, MinKim [15] aims to be low cost and consumes few computational resources.

Regarding driver fatigue detection, Abulkhair et al. [18] propose a system for detecting driver fatigue to take advantage of the use of the driver’s smartphones and its mobility and to avoid the use of larger computers for driver fatigue detection. Patel et al. [19] use a video camera to monitor eye states. Jung et al. [20] developed a method with the use of electrocardiogram analyzing the variability of the heart rate to bypass problems of illumination, common in systems of computer vision. Aiming to perform lane departure detection related to driver fatigue, McDonald et al. [21] developed a vehicle-based approach by applying a Random Forest classifier to the steering wheel angle data. Mehta et al. [22] proposed an approach to detect driver drowsiness using SVM to capture the drivers’ face frames and calculate the EAR Eye Aspect Ratio (EAR), then from a threshold value (EAR=0.25) to infer that the Driver is sleepy. They tested the method with different classifiers. We highlight SVM and Random Forest that obtained an accuracy of 80% and 84%, respectively. Finally, Pauly and Sankar [23] presented a method of detecting drowsiness based on the Viola and Jones method with the use of images from a web camera. His detection used SVM as a classifier for eye blinking. Finally, the PERCLOS uses a limit value of six seconds. Pauly and Sankar showed that 91.6% match with the judgments of that of a human rater.

Finally, the most accepted method of drowsiness analysis is the PERCLOS [24], which uses the proportion that the eyes are 80% to 100% closed in a time interval. However, some authors, such as Kong et al. [25], have used a PERCLOS simplified that uses the percentage of the duration of entirely closed eye state at a specific time interval (1 min or 30 s). PERCLOS based systems have a good acceptance and good correlation with drowsiness. However, among their disadvantages, Stanton et al. [26] mention that the use of a system to identify slow eyelid closure generally requires a restricted field of vision. Thus, the head movements of the driver may require more than one camera. Besides, it may have low efficiency in low humidity environments, as users may be prone to close their eyes slowly and keep them closed for a while, thus mistaking fatigue with humidification, resulting in false positive. It is essential to highlight that we do not found any research about the use of SPC methods in ADAS. Thus we agree that this is our main contribution.

## 3. The Statistical Process Control with EWMA

Statistical Process Control (SPC) is a quality engineering technique that can be used to control and, where possible, make improvements in the production process. The objective of statistical control is to monitor the process to identify sources of variability and, if necessary, to take some corrective action to eliminate the type of event that caused it. We monitor the current state of data distribution accuracy to control this process using variable data. For this, the target value, which corresponds to the desired value for a particular characteristic of a product, is compared to limits indicating its conformity with characteristics of good quality. We perform all this monitoring with the help of control graphs [27].

The control graphs are the main components of the SPC—to allow the identification of the behavior of the process over time or the number of samples and the detection of the incidence of particular causes, we perform this through a history of data. From the identification and detection, it is possible to take actions to prevent and avoid recurrence of the event. This whole process can be performed and controlled in real time. Besides, according to Borror et al. [28], the control graph has the advantage of its operational simplicity and effectiveness in the detection of problems in the process.

The purpose of using statistical process control to monitor the driver is to identify moments of erratic driving, such as actions that can cause accidents. Thus driving should be safe, within certain limits, to maintain the quality of driving. For this, we use a control graph for online monitoring by quickly detecting the occurrence of causes attributable to some event in process changes so that we can take some corrective action before the problem occurs [4].

The control graph represents a quality characteristic concerning the number of samples or time. It has three lines, a call of Central Line (CL) that represents the average value of the quality characteristic and corresponds to the state under control. Two other horizontal lines, called Upper Control Limit (UCL) and Lower Control Limit (LCL), are used to control the range of data variability.

According to Montgomery [4], the control graph works comparing the average of sampled values $\bar{x}$, with the two control limits (UCL and LCL),

$$LCL\leq \bar{x}\leq UCL.$$

If the measure is within limits, the process is under control, and we do not perform any action. Otherwise, when we identify an out-of-control situation, we perform some correction or identify the cause.

**Figure 1:**
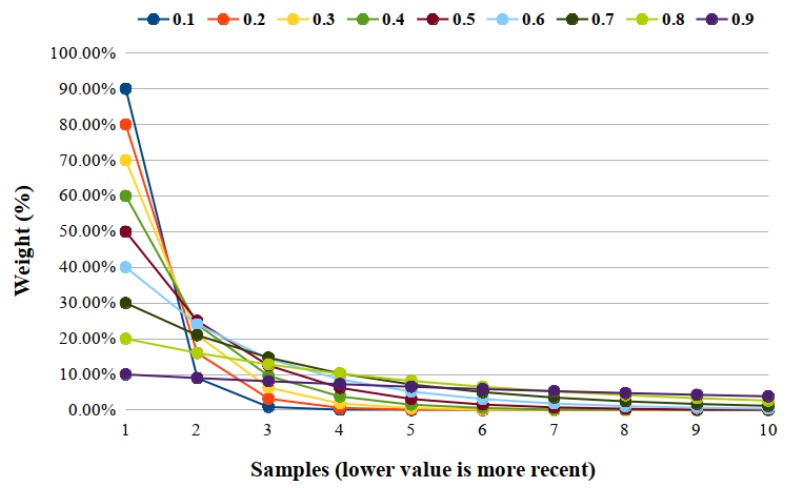
Graphic of the decay of the samples weight from lambda values (λ). Values between 0 and 1.

The statistical measure chosen to analyze and monitor driving data was the Exponentially Weighted Moving Average (EWMA) [5]. We chose the EWMA because it is the faster and more usual SPC method used in industrial control scenarios. We use an EWMA graph when rapid detection of out-of-control situations is required by calculating the time series of measures [29]. In the first step of the EWMA calculation, the measures of the processes are sampled at specific periods and grouped into subgroups of predefined size. We calculate the average and the standard deviation of each subgroup. Then, the EWMA statistic, $z_{i}$ at time *i*, is recursively calculated from the average of the values of the subgroups sampled. We calculate the first value of the EWMA series as the average of the first subgroup. EWMA [5] is given by

$$z_{i}=\lambda \;x_{i}+\left(1-\lambda \right)\;z_{i-1},$$

where i∈N, λ is a constant called decay factor, 0<λ≤1. $z_{i-1}$ is the previous value, so that $z_{0}=\mu_{0}$ and $x_{i}$ indicates the *i*-th sampling. $\mu_{0}$ is the mean of initial samples when the process is in-control. However, since the distribution may not be known, the average of some preliminary data is used as the initial EWMA value, $z_{0}=\bar{x}$, where $\bar{x}$ is the average of initial samples. Figure 1 illustrates an approximation of the value of the weights for several lambda values, calculated by $\left(1-\lambda \right)\;\lambda^{\left(i-1\right)}$.

This factor of decay (λ) allows to adjust the weight of the samples considered in the EWMA and, therefore, to consider more recent samples and to disregard older samples. For example, the graph from Figure 1 demonstrates that higher values of λ, as 0.9, allow a soft decay, thus considering more samples, while low values of λ as 0.2, have a marked decline, where very recent samples are of much higher relevance than the others.

Thus, for our proposal, a smoother decay is more indicated, so the value 0.9 is ideal. However, a fine-tuning with details of the solution is necessary for this. The main detail is the number of samples that are relevant, for a proposal focused on safety in driving, times greater than 1 s can be very large. Therefore, we fixed that samples in a range of 1 s should have a good weight, noting that the most recent should have a slightly more substantial weight.

**Figure 2:**
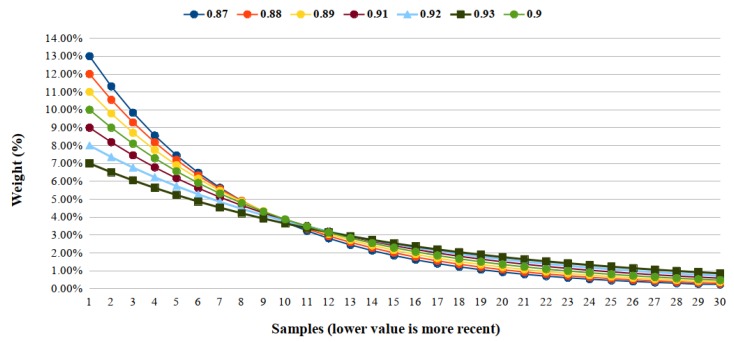
Graphic of the decay of the samples weight from lambda (λ) values close to 0.9. Values between 0.87 and 0.93.

From this, since our solutions have an update rate of 10 samples per second, we consider that 10 samples make up the optimal set of our solution. We calculate the EWMA with values close to 0.9, as shown in Figure 2. We can see that in the tenth reading, the value 0.9 is the one with the highest weight (0.87=3.71%,0.88=3.80%,0.89=3.85%,0.9=3.87%,0.91=3.85%,0.92=3.78%,0.93=3.64%). Despite this, the most indicated lambda value is 0.93, because it is softer than other ones. The graph curve shows that the weight of its most recent sample is quite low, only twice as large as the tenth sample. So 0.9 is the best value for our solution. Nevertheless, we can use close values since the weight of each sample is very close and would generate a few differences.

In this way, the control graph can be defined with the following limits

(1) $$\begin{array}{ccc} UCL & = & \mu_{0}+L\;\sigma \;\sqrt{\frac{\lambda }{2-\lambda }} \end{array}$$

(2) $$\begin{array}{ccc} CL & = & \mu_{0} \end{array}$$

(3) $$\begin{array}{ccc} LCL & = & \mu_{0}-L\;\sigma \;\sqrt{\frac{\lambda }{2-\lambda }} \end{array}$$

where $\mu_{0}$ is the mean of initial samples when the process is in-control, *L* determines the width of the control limits and σ is the process standard deviation, $\frac{\lambda }{2-\lambda }$ is the standard deviation component of EWMA statistics and *L* is a factor that allows a greater opening of the limits, usually 2 or 3. Thus, the *L* allows the control of the limits.

The EWMA calculation allows incorporating information from all subgroups of previous measures, with weights that increase the relevance of the last calculated sub-group. Thus a control decision is made based on the information from the previous subgroups and the current one.

EWMA is considered to be a quasi-non-parametric procedure, free of distribution, as Borror et al. [30] demonstrates. Moreover, Hunter [5] shows that the EWMA allows later samples to have larger weights. This feature is unusual for the proposal since it allows to consider subgroups with weights. Due to these characteristics, EWMA allows the sample to be more recent and the most relevant for identification.

## 4. Vehicle Drivers Monitoring through EWMA

The use of SPC in driver assistance systems is intended to facilitate the detection of errors and thus assist in the process of monitoring driving through EWMA applied to drive data. In this section, we present the different methods proposed for the monitoring of conductors, using the EWMA. We apply these methods to the following problems: i. Lane departure detection; ii. Sudden driver movement detection. iii. Driver fatigue detection;

### 4.1. Lane Departure Detection

We develop a method considering wheel data collected through accelerometer and gyroscope variables. The gyroscope decreases the rate of false positives because this sensor allows obtaining the acceleration at a specific moment without any calculations by the system. Thus, the acceleration of motion also becomes better evaluated, allowing better detection at high speeds. Tests involving only the accelerometer analyze more the amplitude of the movement than its acceleration, making this sensor necessary to increase the range of incorrect situations detected.

In this way, the identification of the dangerous movement of lane departure necessitated obtaining the data of the axes *X* and *Y* of the accelerometer (acceleration in ms2) and of the gyroscope (angular speed in ∘s) connected to the steering wheel, data processing and then the application of the EWMA control graph with λ=0.9 and factor L=3 in the data for the identification of events. The application of the EWMA graph first required a specification of steering wheel values, which we call calibration, to identify dangerous movements in lane departure. The values of the specification depend, as mentioned, mainly, on the speed of the vehicle. When a move that does not meet the specification occurs, the EWMA quickly detects it. 30-s specifications were generated for each direction of rotation of the steering wheel, clockwise and counterclockwise, each to detect events in its direction when we apply the EWMA to the *X* axis and both directions when we apply the control graph to the *Y* axis. We generate eight control graphs, one for each of the two calibrations (calibration counterclockwise and clockwise) for each axis (*X* and *Y*) and each of the two sensors (accelerometer and gyroscope).

The final result of the detections is the combination of the results of the two sensors generated by each of the calibrations. This combination of sensors is given by

(accX∪accY)∩(girX∪girY)

so that the sets of points accX and accY detected as lane departure along the *X* and *Y* axis, respectively; and the sets of points girX and girY detected by the *X* and *Y* axes of the gyroscope, respectively. In this way, the control graph can identify out-of-control conditions when points are out of control limits, LCL e UCL.

We can obtain the angular position data through CAN bus of the car, in case of vehicles with electric assist direction. However, the application of EWMA will be similar. How we identify sudden movements is that the solution locates the lanes on the road without any environment perception equipment, like a camera. However, the results (Section 5) present a high number of false positives and negatives. This result motivates the proposal of sudden movements detection (Section 4.3), based on EWMA, and the driver fatigue detection (Section 4.4), based on image processing. Used together, they presented better results than the previous one. However, in order to provide a more complete solution we also consider the lane departure detection by monitoring the road with a camera (Section 4.2), because using only acceleration and yaw rate data would not be sufficient to detect lane departure due to the robustness issue to the disturbance and the unknown relative lane position.

### 4.2. Lane Departure Detection by Monitoring the Road with a Camera

**Figure 3:**
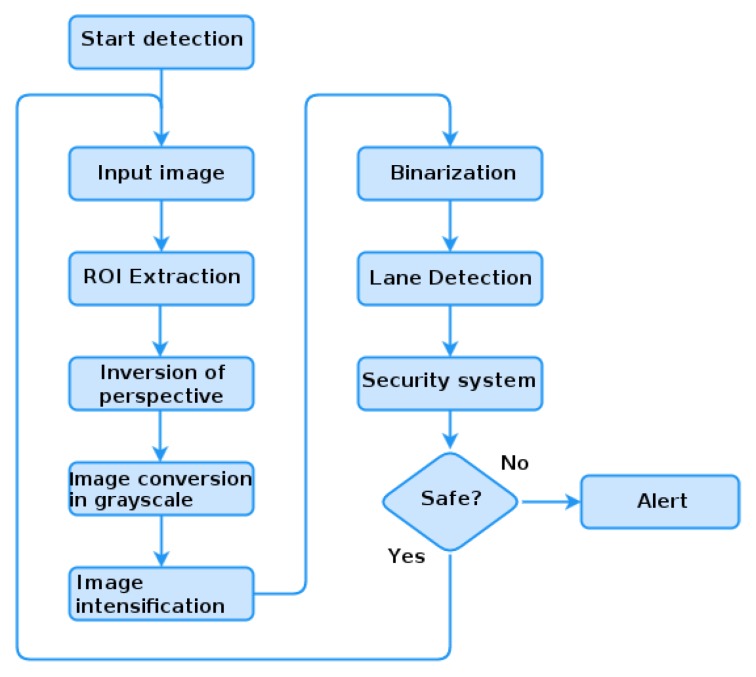
Lane detection flowchart.

This lane detection technique is intended to analyze the position of the vehicle concerning the lanes and thus indicate that the vehicle is moving out of lane or even if there was lane departure. For this purpose, we use several image processing techniques in order to be able to design efficient detection. The detection process used is illustrated in the flowchart of Figure 3.

The flowchart, for lane detection, consists of the following steps:

1. We extract the region of predefined interest (ROI) of the input image, on this image we apply the inversion of perspective by the technique *bird’s-eye image* [31] creating an image with parallel lanes. This step is necessary since the lane identification technique requires that they be parallel to estimate their positioning;
2. A filter is used to intensify the marking of the lane [32] through a precomputed pixel width of the grayscale image, in the image line. The filter intensifies the pixel value of the image lane to another intensity.
3. With the intensified image, a threshold is adopted in order to make the binary image.
4. The pixels that are above the threshold receive the value 1 (white color) represented by the lane and 0 (black color) the rest of the image. The distance transformation formula is applied using as a metric the Euclidean distance in the binarized image [31]. This distance transformation is used to find the distance from the current pixel to the nearest white pixel. From this distance, we generate a gradient image where the darker regions are the lanes.
5. With the lane detected, the road is divided into two sectors, the left lane and right lane. The median of these lanes is the center of the road. From the center of the image (center of the vehicle), we can obtain the variation of the position of the vehicle on the road. With each limit, left and right, as 100%, we consider a position higher than 60% as invading another lane. We detect a zigzag movement by analysis in a time interval.

**Figure 4:**
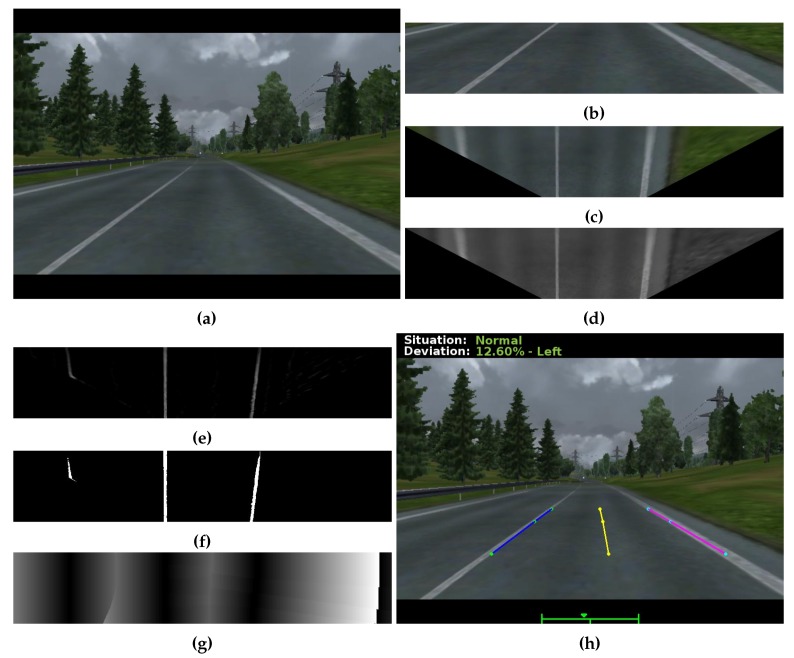
Lane detection on one of the *frames* experiments. (**a**) the input image; (**b**) the predefined Region of Interest (ROI); (**c**) ROI with the technical application *bird’eye* [31]; (**d**) ROI in grayscale; (**e**) the intensified image; (**f**) the binarized image; (**g**) the transformation of distance; and (**h**) the end result of lane detection.

The application of the detection process used can be seen in Figure 4.

It is worth noting that this method of lane detection has some limitations; as an example, we can list: i. vehicles within the region of interest may impair the binarization phase of the image; ii. although the method has efficiency at night, vehicle headlights in the opposite direction can generate false positives; and iii. if one of the road lanes is erased or covered up, such as by land, detection is impaired. It is essential to highlight that, once this strategy does not use EWMA, we do not use it to evaluate our system (Section 5). The system could use the lane information generated by this strategy to improve the robustness of the system. However, we propose to avoid the frontal camera to keep the system cheap and with a viable time complexity to execute in an embedded system.

### 4.3. Sudden Movements Detection

The detection of errors caused by sudden movements aims to identify two types of event: i. sudden movements in the steering wheel that cause lane deviation; and ii. sudden movements on the brake pedal. These events are dangerous because they can cause some accidents and even indicate that the driver is drowsy.

From the EWMA control limits, it is possible to identify that there were sudden movements. For sudden movements in the steering wheel that generate lane deviations, we generate specifications of accepted values, which we call calibration. This specification depends on the speed of the vehicle, and the turns that go counterclockwise.

When a move that does not meet the specification occurs, the EWMA quickly detects it. Detection occurs through the application of the control graph to accelerating data (ms2) of the *X* axis of the accelerometer. The pedal used is the brake pedal. We calibrate it, and the data analyzed represent the distance between the pedal and its support, in centimeters, so that it is 7 cm when it is at rest.

Due to problems in the accelerometer and potentiometer signals, such as noise and inaccuracy, we apply the Low Pass Filter with Moving Average:

$$new_{i}=\frac{\left(sample_{i}+sample_{i-1}+sample_{i-n-1}\right)}{n},$$

where i≥n is the index of the new sample, starting at the *n*. This filter is the average of the last signal samples *n*, as *n* being the size of the window. This moving average must be adjusted to each sensor, thus not having an ideal value to obtain a better signal. Tests should be made to verify the noises’ remotion without generating significant delays in the signal. Each sensor used a window size that best suited the signal type. We define this window from the analysis of the signal with values that ranged from 7 to 2, in this way, we chose the sizes: 2 for the accelerometer and 3 for the potentiometer.

**Figure 5:**
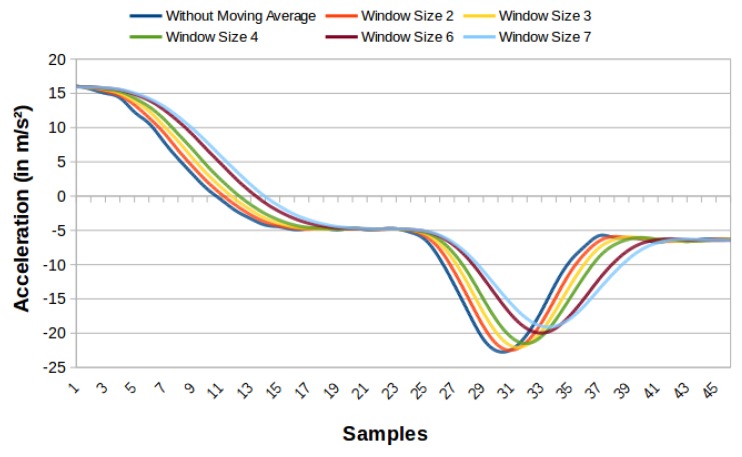
Comparison graph of the accelerometer reading on the steering wheel without moving average and moving average with windows of sizes 2, 3, 4, 6 and 7.

**Figure 6:**
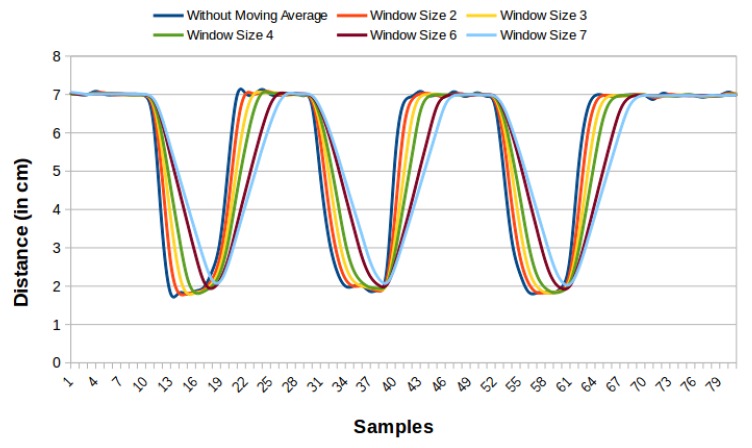
Graph reading comparison of the potentiometer reading on the brake pedal without moving average and moving average with windows of sizes 2, 3, 4, 6 and 7.

We obtain the values used in the moving average for the accelerometer, and the potentiometer through tests. We verify the best signal generated for each sensor. The graphics in Figure 5 and Figure 6 present some readings with different window sizes. We can see in Figure 5, that the curve without moving average presents some noise, being a little soft, whereas the curve with the size two window has this problem reduced. Larger window sizes, while showing smoother curves, add a considerable delay, making them less attractive to use. Therefore, size two was considered better for the accelerometer. For the potentiometer (Figure 6), the curve with window size 2 softens the signal. However, the signal needs to be even smoother. Thus, window size three was chosen, even adding a small delay because it has a smoother curve. Larger window sizes generate a much more significant delay with few benefits in the curve.

### 4.4. Driver Fatigue Detection

We detect driver fatigue through driver characteristics that are extracted using image processing, pattern recognition, and SPC techniques. This method has as input only the images acquired by the camera facing the driver’s face. Camera image processing provides driver related information. This information is the face direction (front, left or right) and eye condition (open or closed) in instant data.

**Figure 7:**
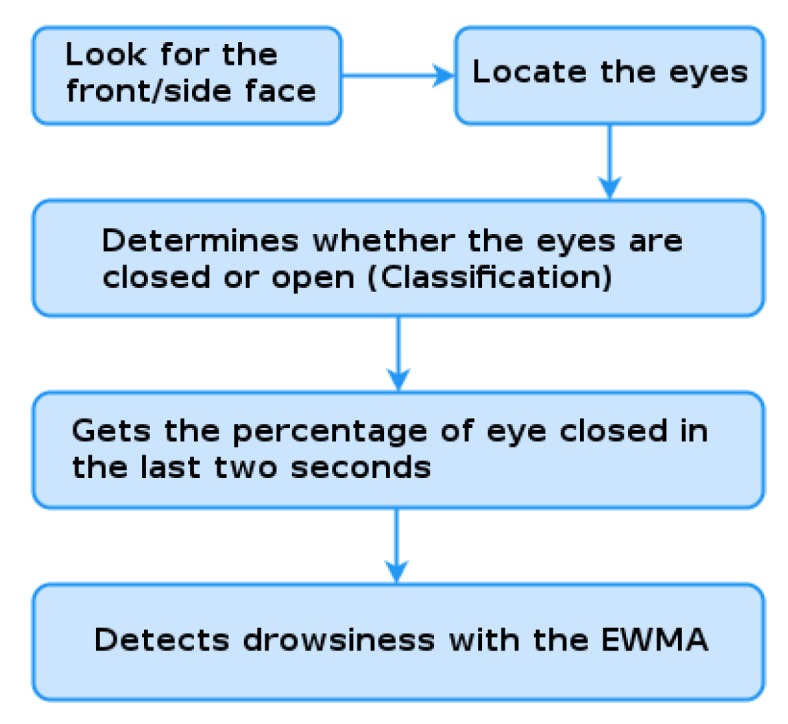
Driver fatigue detection diagram.

We divide the driver fatigue detector into three stages: i. face detection; ii. eyes classification; and iii. application of the EWMA control graph to the closed eye percentage of the last two seconds. The steps for detecting driver fatigue are illustrated in Figure 7.

Face detection is performed using the method proposed by Viola and Jones [33] with the increases proposed by Lienhart and Maydt [34]. In the first, Viola and Jones use machine learning to detect objects in images, dividing the base into two parts, one containing the object and the other not, to extract features that differ from the two parts. The proposal of Lienhart and Maydt uses more characteristics in the learning frame employing rotations in 45° in the classifier training.

In our proposal, we use two classifiers, one trained to detect frontal faces and another trained to detect lateral faces. We train the side face detection classifier with right-sided profile faces, so to detect left-facing faces the image is mirrored before the search process. The first step of the algorithm is to search for the front face, if found, the algorithm passes to the next step, the classification of the eyes. If the front face is not detected, the algorithm searches the side face (left and right) and then it processes the next image.

We perform the classification of the eyes as follows: i. Locate the eyes: during software initialization, the location is made using a trained classifier to locate eyes using techniques used for face detection; ii. The location of the eye is done through template matching, using a template saved at startup. iii. The classification determines if the driver’s eyes are open or closed at that moment. We classify partially open eyes as open. The classification is made using three Support Vector Machine (SVM) classifiers, trained with a base 4000 open eyes and 4000 closed eyes, these classifiers receive as input data taken from the following image descriptors: Local Binary Pattern (LBP) [35], Local Ternary Pattern (LTP) [36] and Histograms of Oriented Gradient (HOG) [37]. By classifying the eyes in a sequence of images, it is possible to detect the driver’s blinks and also to measure the duration of each of them. The driver fatigue detector extracts the percentage of closed-eye time per reading at each 2 s. We use this percentage as input in our EWMA control graph. In this case, we have only one graph.

For monitoring the driver’s attention, we use the EWMA control graph with the eye closed by reading using λ=0.9 and factor L=3, indicating moments of driver fatigue. An observer validates fatigue moments. In addition, an audible alert may be issued to indicate to the driver that he is drowsy and that he must resume attention.

## 5. Evaluations and Results

In this section, we present the results of the evaluations performed for the proposed methods. The objective of the evaluations is to identify the efficiency of the methods and the prototype in a simulated environment.

**Figure 8:**
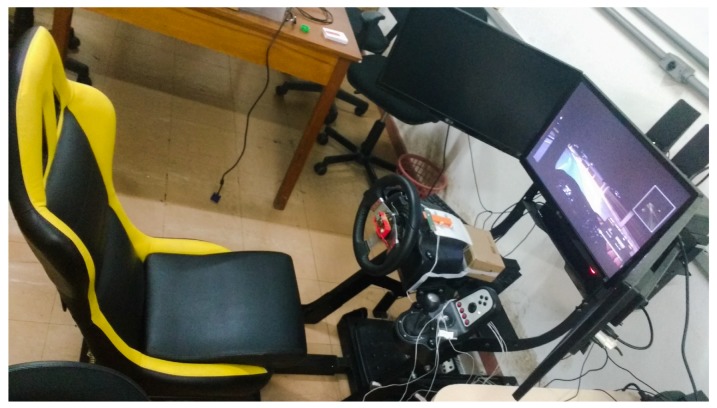
Driving simulator.

Because it is not safe to conduct tests related to driving errors on the road, we use a vehicle emulator environment to conduct experiments. Some of the main advantages of its use are experimental control, low cost, efficiency, security, and ease in data collection. The emulator used consists of a computer, a set of monitors, a realistic cockpit, a steering wheel, manual gearbox and clutch pedals, brake and accelerator [38] (Figure 8).

Some researchers such as Auberlet et al. [39] and Mayhew et al. [40] perform the validation of the use of emulators to create a real-world environment because this environment seems to include limitations, as the driver does not realize the real risks about real driving. However, Mayhew et al. [40] state that “(…) collectively, the results of the concurrent and discriminant validity studies support the use of the simulator as a valid measure of driving performance for research purposes”. Thus, the tests were done using the Euro Truck Simulator 2 (Euro Truck Simulator 2, Last Access, April 2019: http://eurotrucksimulator2.com/) which according to Lee et al. [41], realistically imitates the driving process, and according to the parameters of each scenario. Besides, an automatic transmission was used in the experiments to avoid noise caused by the change of gears in the obtained data.

### 5.1. Lane Departure Detection

In this section, we present our initial evaluation of the lane departure detection methods with two sensors. The objective is to show that by using two simple sensors, we can detect the drivers’ behaviors.

#### 5.1.1. Experiment Setup

To evaluate the lane departure detection technique, we have developed a functional prototype for lane departure detection. The first version (used in the first scenario evaluated) considers a tablet, the GT-P4500, which has 1 GB of LPDDR2 memory, 32 GB of internal memory and a 1 Ghz Dual-Core ARM Cortex-A9 processor, was coupled to the tablet with the function of obtaining the values of the three axes, *X*, *Y* and *Z*, of the accelerometer in ms2 and of the gyroscope in ∘s. This device has a capacitive type accelerometer, the KXTF9 manufactured by Kionix, and a gyroscope MPL Gyro.

**Figure 9:**
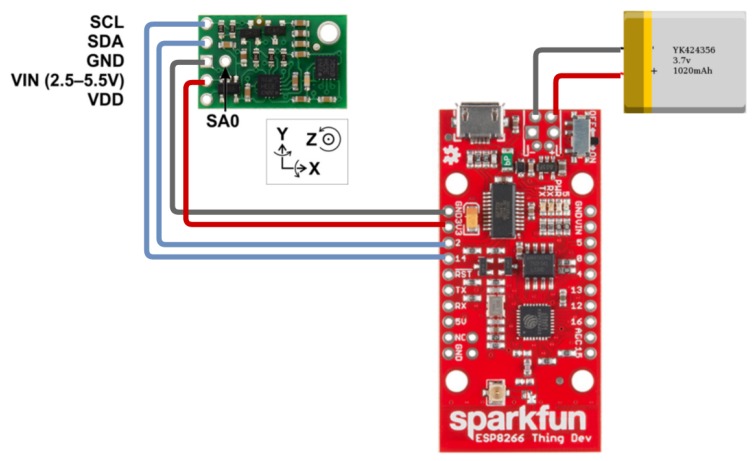
Connection diagram of the prototype components (module ESP8266, IMU unit and battery).

The second and more sophisticated version (used in the second scenario evaluated) obtains the data using an Inertial Measurement Unit (IMU) [42] with an accelerometer, gyroscope, and magnetometer connected to a microcontroller ESP8266 [43] with Wi-Fi interface. We install the microcontroller with the IMU in the center of the steering wheel. Figure 9 illustrates the wiring diagram of the microcontroller to the IMU unit and the battery. It shows that the connection between the microcontroller and the IMU takes place through the respective pairs of pins: 3V3/VIN, GND/GND, SDA(2)/SDA, SCL(14)/SCL. The connection between the microcontroller and the battery is via the JST connector. In order to send the code to the module, we use an FTDI Serial USB RS232 Converter, where the pins of the microcontroller were connected to the converter as follows: GND to GND, NC to CTS, 3V3 to VCC, RXI to TXO, TXO to RXI and DTR to DTR. This connection was not included in the figure since it is a connection used only for recording the source code of the prototype in the module.

The developed prototype uses a web service on the Wi-Fi module and an Android application to obtain and analyze this data using EWMA graphics. It was developed to take advantage of the processing of mobile devices that are common to users and thus to avoid increasing the cost of the prototype to end consumers. In addition, it has a reduced size, about 22mm×66mm×42mm. The application is connected to the module’s Wi-Fi network and calibrated by the user from the steering wheel rotations to the two sides for 30 s. The angle to the rotation for each side varies according to the automotive system and its sensitivity, being an angle smaller than one that could generate a lane departure by sudden movement.

We perform the lane departure monitored by the prototype with EWMA under the data of the *X* and *Y* axes of the accelerometer and gyroscope. First, the application begins to consume the data from the web service and performs a calibration where the conductor rotates the steering wheel to both sides in ≈$30^{\circ }$. We use this value because in the tests carried out. It proved to be the “limit” angle that did not generate speed range between ≈40 and ≈60 Kmh. We store this calibration in the application for future routes, and we can recalculate if necessary, this prevents the calibration of each route. For the calculation of EWMA control limits we use factor L=3 and λ=0.9. We chose these values because they guarantee a higher weight to the most recent readings, but with a soft decay, as presented in Section 3. However, to ensure a more robust system, these values can be reconfigured. After calibration, the system begins to monitor the data it obtains from the web service and, in case of an out-of-control point, as caused by an incorrect movement, the prototype issues an audible alert.

Finally, for both the accelerometer and the gyroscope, the sensor can add Brownian noise [44], obtaining an offset in the expected reading. Besides, reading the sensor data is converted into an electronic signal subject to electronic noise, generating an unexpected departure. For this reason, it is necessary to apply filters and calibrate the data so that it is possible to obtain a data set with greater precision. We used an Average Resting Calibration and the Low Pass Filter with Cumulative Average to mitigate error in sensor readings. For the average resting, we used samples obtained in the range of 30 s, thus, with the steering wheel and sensor at rest, samples were obtained that were used for an average considered as zero reference value. The low-pass filtering consisted of reducing the set of measures by a cumulative mean. For this task, we use a regular number of samples, since using many samples could lead to data loss, while a few samples could remove the noise. This accumulated average was calculated every three samples, because for a proper analysis, some readings per second are necessary, and we were able to register, without errors, 10 readings per second. For the beginning of the tests, an alignment was made to establish the reference attitude. We perform this alignment with the technique “gyro-compassing” [45].

#### 5.1.2. First Scenarios Evaluated

In order to validate the proposed methods, we evaluated the rate and number of identified events, false positives, and false negatives. These experiments were carried out in a controlled manner in a path of the driving emulator presented at the beginning of this section. In the scenarios used, the driver made deliberate errors at some points along the way. The movements were analyzed and validated by the observer for verification with the control graph. We evaluate these movements through the movement of the steering wheel correlated with the position of the vehicle in the lane from the observation of this position.

**Figure 10:**
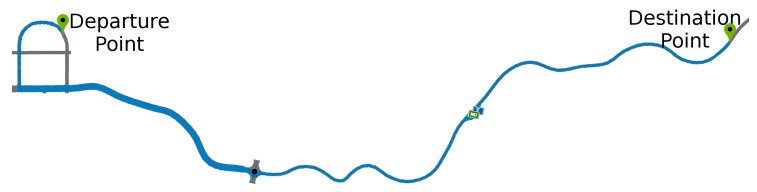
Test route in driving simulator.

The test route used (Figure 10) was chosen to include several driving situations, such as straight road, corners, and curves, stretches of double lanes and lanes with opposite directions. An observer evaluated all the experiments in order to validate lane departures for comparison with the graph. The experiments were performed by adult drivers under normal conditions and with a driver’s license and repeated 25 times. The drivers or tests have the following specifications:

- Each driving lasted about 5 min.
- The drivings followed a predefined route;
- The driver kept the vehicle speed around 40 kmh.
- The drivers are informed that they must make some mistakes at certain times.
- Sudden movements were performed each 40s, approximately.
- The first four movements were counterclockwise, and the other four were clockwise, totaling eight movements.
- The lane deviations were observed, only large deviations were accepted where the entire vehicle crossed the lane limits, invading the other lane, characterizing a lane departure.

The drivers were without signs of drowsiness or any other abnormal condition because it was not our goal to assess the causes of lane departures.

In order to apply the EWMA control graph to the samples, specifications were generated using the steering wheel movements within a standard pattern (movements that do not generate large lane deviations—lane departures) for the speed defined in the experiments, 40kmh. This specification will be called the EWMA calibration. Two types of the specification were generated, with counterclockwise rotations and clockwise rotations, each with data of 30 s of duration. We use the data obtained in the conduction tests in the control graph with the calibration data, thus generating two graphs for each group (*X* axis and *Y* axis data) of samples. Each graph is referring to calibration and thus to a direction of rotation (counterclockwise and clockwise). Each graph has as objective to identify errors related to turns in the direction of rotation of the calibration. However, the *Y* axis allows the identification in two directions.

As seen in Section 3, the EWMA definition has a constant λ and the limits of the control graph have a factor *L*, as defined in EWMA equations. We chose factor L=3 for the experiments so that the limits were slightly away from the center line, avoiding a high false positive rate. We chose λ=0.9 so that several recent readings to weight the results. A value below λ would make only the very recent readings have weight, as presented in Figure 1. For example, with a λ=0.9 the first reading would have a 0.1 weight and the tenth 0.03 while with λ=0.2, the first reading would have 0.8 weight and the 10th $8.192\times 10^{-8}$, an extremely insignificant value. The vehicle speed was maintained around 40kmh because in Brazil this is the speed in collecting ways (streets that allow access to and exit of arterial roads, usually with traffic lights and allowing movement within a region of the city). Finally, the refresh rate was 100 ms, since it was the rate at which the application gets data without loss of performance or accuracy. Table 1 shows a summary of the parameters used in the experiments.

**Figure 11:**
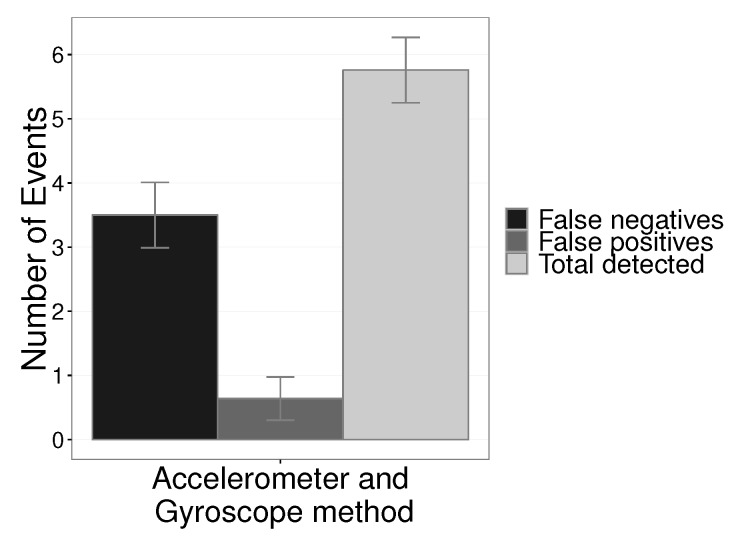
Number of detected EWMA application events on accelerometer and gyroscope.

Figure 11 shows the number of events detected in absolute numbers with a combination of the EWMA application results in the accelerometer and gyroscope. The results present the average values with a confidence interval of 95%. The number of errors that could be detected was 8. The combination of the data from the two sensor axes had a good part of the eight errors detected with low false negatives ≈5.76 and only ≈0.11 false positives.

**Figure 12:**
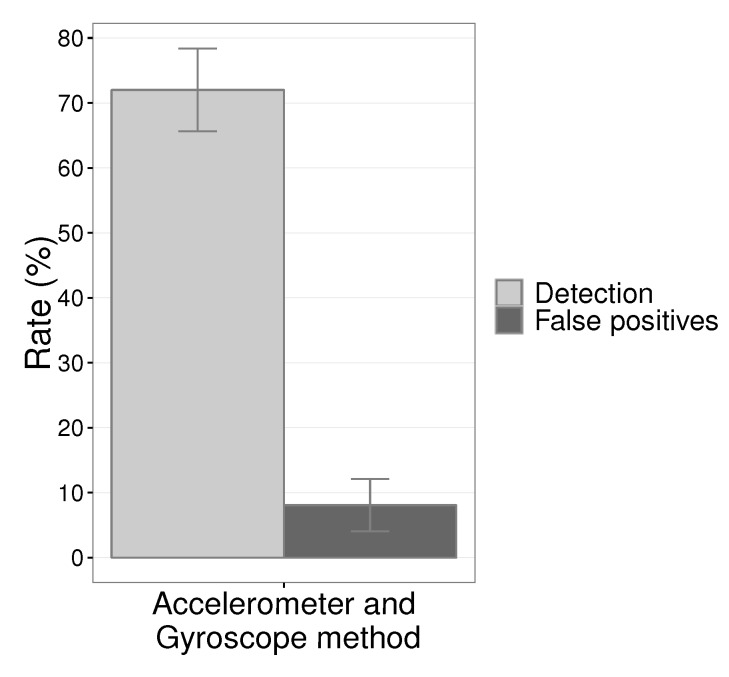
Detection rate and false positive rate with the combination of the results of the two axes, *X* and *Y*.

Figure 12 shows the results of detection rate and false positives with the combination of the results of the axes *X* and *Y*. The detection rate was 72% with a low false-positive rate of only 8.07%.

Table 2 illustrates the application of confusion matrix on test data. We can observe that the ability to correctly predict what it values is 62% (sensitivity), showing to be able to recognize essential cases in 98% of the cases (precision) and thus the precision of the test is the F1 Score which was 76%.

This efficiency is a feature of the EWMA control chart that allows efficient and rapid detection of out-of-control events in a process. Testing in a controlled environment is one factor that may have contributed to high efficiency.

**Figure 13:**
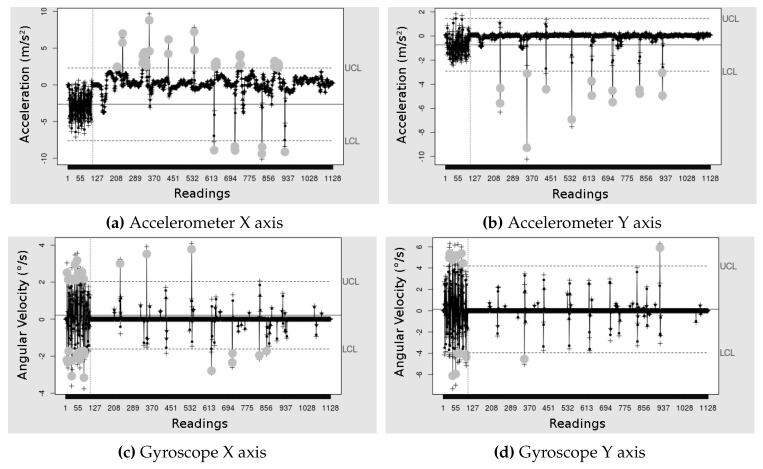
EWMA control graphs applied to the accelerometer data, in m/s${}^{2}$, and the gyroscope, in °/s. Calibration with clockwise rotation data.

**Figure 14:**
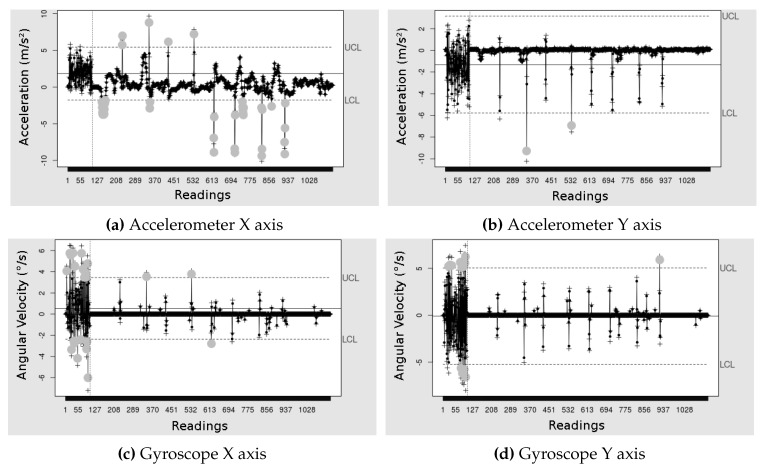
EWMA control graphs applied to the accelerometer data, in m/s${}^{2}$, and the of the gyroscope, in °/s. Calibration with rotation data counterclockwise.

Finally, the Figure 13 and Figure 14 show the EWMA graphs of one of the test conductions. On the figure, each “Reading” is a sample and then the abscissa values are the samples. The graphs with the axes *X* and *Y* of the accelerometer, Figure 13a,b show all eight errors being detected, gray dots, with few false positives in the limit UCL of the graph of the *X* axis. While the EWMA with the axes *X* and *Y* of gyroscope, Figure 13c,d did not identify all the errors, so that the *Y* axis detected only two of the errors. However, the combination of the sensors allowed the detection of most errors.

The graph with the *X* axis of the accelerometer, Figure 14a shows all eight errors being detected (gray dots), with few false positives in the limit LCL, however, the graph of Figure 14b with the *Y* axis detected only two errors. While the EWMA with the axes *X* and *Y* of the gyroscope, Figure 14c,d did not identify all the errors, so that the *Y* axis detected only two of the errors. The combination of sensors enabled detection of most errors.

Our proposal used the union of the *X* axis and the *Y* axis of each sensor, since they are complementary, and the intersection of the two sensors so that the detection was more faithful to the reality when being detected by the two sensors. However, because the sensors intersect, some events can only be detected by the accelerometer (amplitude of movement) and not by the angular velocity (gyroscope) or otherwise, thus reducing its efficiency concerning the gyroscope only. A different combination of the detections of each of the sensors can result in higher precision.

#### 5.1.3. Second Scenario Evaluated

**Figure 15:**
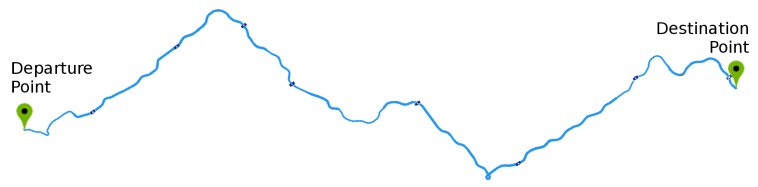
Route for testing with the prototype in the driving emulator.

Figure 15 shows the tested route. The experiments consisted of a scenario where the driver committed some lane departures during the course. We analyze the driver by an observer and by the system, in this way, it was possible to analyze when the prototype emitted a correct alarm. We set the period for each record to 30 min. The experiments were performed by adult drivers under normal conditions and with a driver’s license and repeated 13 times without constant speed and six times with maintained speed close to 60 kmh and the results presented average values with a confidence interval of 95%.

Thus, we performed experiments in two ways. In the first one, the drivers had to keep the vehicle, as far as possible, 60 kmh and in the second one, we did not define the speed. Thus, they obeyed the following items:

- 30 min of driving.
- Performing 20 lane departure movements as defined in the proposal.
- The movements were to the two sides, left and right, being thus from turns in the steering wheel in two directions: anti-clockwise and time.

**Figure 16:**
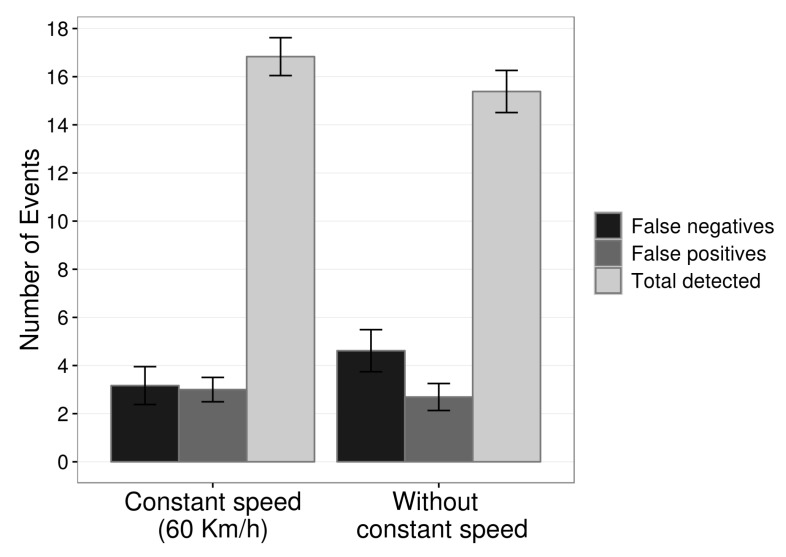
Number of events detected in absolute number by prototype with constant speed and without constant speed.

The graph of Figure 16 shows the complete number of results of detections with constant speed and without constant speed. It is possible to observe that at constant speed the number of events detected was slightly higher than without a constant speed, ≈16.83 against ≈15.38. The number of false positives was very close, ≈3 with speed maintained close to 60 kmh and ≈2.69 with speed varying to any value. Finally, the false negatives were a bit lower with constant speed, getting slightly above 3, ≈3.16, different from the other approach that reached ≈4.61.

**Figure 17:**
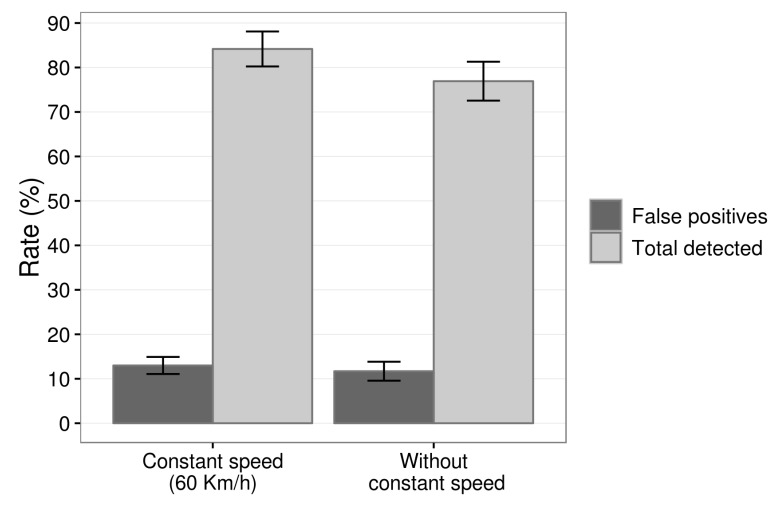
Detection rate and false positive rate of prototype with constant speed (≈60kmh) and without constant speed.

We divide the percent results into two parts, first the results without the control of the vehicle speed and then with speed maintained close to the 60 kmh. The graph of Figure 17 presents the detection and false positive rates of the proposed system with a constant speed close to 60 kmh and without speed control, the driver can drive the vehicle at any speed, normally between 40 kmh and 80 kmh. The application of the EWMA control chart on the data obtained by the microcontroller located on the steering wheel ensured a detection rate of 76.92% with 11.70% and false positives without constant speed. We calculate the percentage of false positives from the sum of the total expected events during driving plus total false positives.

Besides, Figure 17 illustrates the detection rates and false positives of the prototype when drivers have maintained their speed, as close as possible to 60 kmh. We can see that the detection rate reached a value, 84.16%, and 12.98% of false positives. We calculate this percentage of false positives as the previous one.

The comparison between the two forms, constant speed, and no constant speed, indicates that the method has a significant gain when we consider the speed of the vehicle for calibration allowing to control it during driving. Despite this gain, the system had a reasonable detection rate for “more real” drives, where the speed does not remain constant. Besides, a part of the false positives occurred in moments where the vehicle made sharp curves with low speed, like when leaving the garage. However, we consider these false positives because it is a system directed to real environments.

Table 3 illustrates the application of the confusion matrix on test data with constant speed and without constant speed. We can see that the system can correctly predict the condition evaluated in 84% of the cases against 76% tests without a constant speed. Recognizing relevant cases in 86% in both cases, the precision of the tests with constant speed and without constant speed is 85% and 81%, respectively.

### 5.2. Driver Fatigue and Sudden Movements Detection

**Figure 18:**
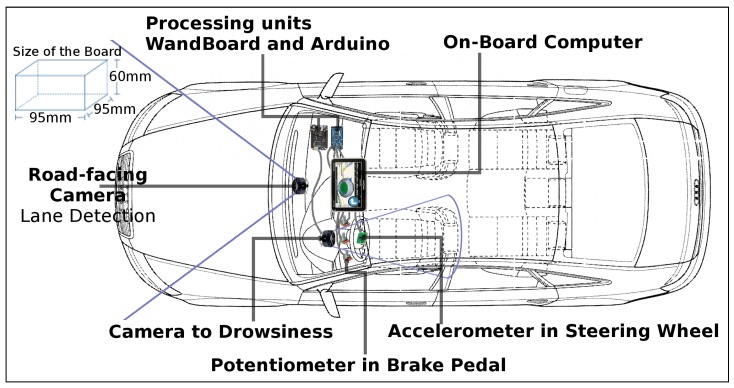
Positioning conceptual the components of hardware of the prototype inside a vehicle.

After the first evaluation, we compose a complete evaluation scenario by considering different sensors and elements in order to identify the driver behaviors. Thus, we execute together with the driver fatigue detection and sudden movements detection with sensors in the steering wheel and a brake pedal. To perform this evaluation, we developed a functional prototype for comprehensive driver monitoring. The prototype consists of a video camera positioned in front of the driver connected to a Quad Wandboard board [46] to capture information from the driver, through his face. In the experiments, we used a 15-megapixel camera, Full HD 1080p, model Logitech C920. We also consider a capacitive accelerometer, model MPU6050 of IMU GY-88, in the steering wheel connected to a Mega 2560 Arduino to capture the movements of the steering wheel; and a rotary potentiometer 10 KΩ on the brake pedal connected to an Arduino to capture the pedal data. Figure 18 illustrates how the positioning of the hardware inside a vehicle could be. In the proposed positioning, we can only vary the location of the processing units. We cannot change the position of the onboard computer and sensors and cameras. The onboard computer needs to be close to the driver so that the driver can hear and visualize the warning signs clearly and have easy access to him to perform other activities of his interest. The road facing camera is a lane departure detection method based on image processing. This technique goes beyond the scope of the article, thus it is not evaluated.

**Figure 19:**
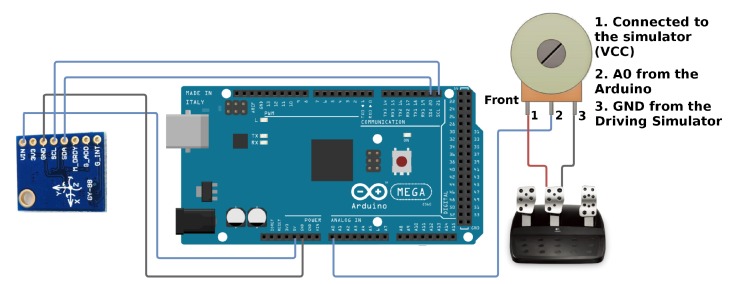
Connection diagram of the Arduino to the accelerometer and potentiometer.

Figure 19 illustrates the connection diagram of the Arduino with the connection between the Arduino and the IMU unit (with accelerometer) located on the steering wheel and the potentiometer located on the brake pedal.

The system meets certain requirements mentioned in [47], acceptable performance when run in real time, ≈20 FPS with a Wandboard and ≈10 readings per second with the Arduino; low cost, around US$30,000; with a reduced size, approximately 95mm×95mm×60mm; and low power consumption and flexibility.

**Figure 20:**
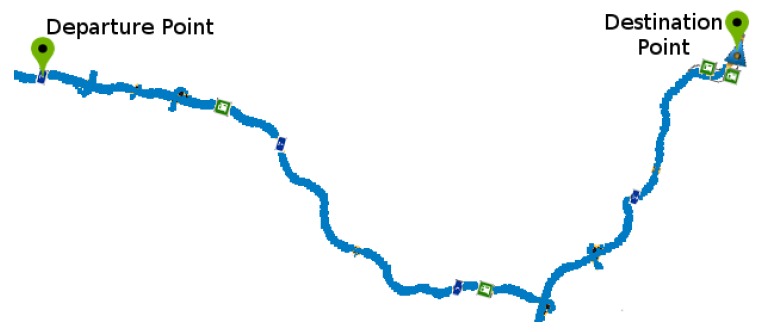
Test route in the driving simulator.

We evaluated the driver fatigue detection and the detection of sudden driving movements together. Separated monitoring leads to a non-satisfactory answer as to the inference about errors made by the drivers. For this evaluation, a driving simulator highway, previously presented, was used. The results presented values with a confidence interval of 95%. The test route considered is illustrated in Figure 20. During the tests, the driver started the vehicle and followed the defined path.

The experiments were carried out by six adult drivers, all males aged 20 to 27 years (mean ± standard deviation (SD): 24±2.8 years), under normal conditions and with a driver’s license in a controlled environment and with the following specifications:

- The driver was asked to keep the vehicle speed around 80 kmh.
- The driving lasted 10 min.
- In the first 5 min, the driver was instructed not to make mistakes and to pay attention.
- After 5 min of driving, the driver was asked to simulate driver fatigue, such as blinking slower and longer. An observer assessed this driver fatigue.
- The driver performs an event at each ≈30 s. We use three types of events:
  1. driver fatigue followed by sudden movements on the steering wheel counterclockwise.
  2. driver fatigue followed by sudden movements on the steering wheel clockwise.
  3. driver fatigue followed by sudden braking, simulating a moment where the vehicle approaches some object and needs to brake to avoid a collision.

Drivers were in normal condition and simulated drowsiness, for example with slower actions and lack of attention, such as closing their eyes for a few milliseconds. Our team evaluated these actions and validated by results.

Due to problems in the accelerometer and potentiometer signals, such as noise and inaccuracy, a low-pass filter of the moving average type is applied. Each sensor used a window size that best suited the signal type. We define this window from the analysis of the signal with values that ranged from 7 to 2. In this way, we chose the sizes: 2 for the accelerometer and 3 for the potentiometer.

The Wandboard microcontroller processes and obtains data from the camera positioned in front of the driver and provides:

- The total number of blinks.
- The number of blinks in the last 20 s.
- The percentage of time the closed eyes at each reading (2 s).
- The percentage of time that the closed eyes in the last 50 s.
- The face position (front, right, left, or distracted).

Arduino microcontroller, on the other hand, retrieves and processes data from:

- Accelerometer (*X* axis) on the steering wheel, in ms2.
- Potentiometer on the brake pedal, in cm. Possibly varying from ≈7 (resting) to ≈1 (pressed).

We apply the EWMA control graph in the samples of each method used as follows:

- Steering wheel monitoring: We generate a specification (calibration) with movements that are not incorrect for the speed of 80 kmh. When a move that does not meet the specification occurs, the EWMA quickly detects it. Detection occurs by applying the graph to the *X* axis acceleration data of the accelerometer located on the steering wheel.
- Brake monitoring: Uses a specification as made for the steering wheel. The data represent the distance between the pedal and the pedal holder, in centimeters, so it is 7 cm when resting. In case of incorrect movement, EWMA detects incorrect moments.
- Monitoring of driver fatigue: The EWMA was applied to the percentage of closed-eye time per reading (2 s) to monitor the driver fatigue. According to the conduction samples, the EWMA generates the limits that allow identifying the driver fatigue.

For the parameters used, we chose factor L=3 so that the boundaries were slightly away from the center line, avoiding a high false positive rate. In order for several recent readings to weight the results, we chose λ=0.9. The vehicle speed was maintained around the 80 kmh because in Brazil this is speed in fast traffic routes (roads with different lanes, without traffic lights, without pedestrian traffic and with great extension) and stretches of highways (paved roads). In this way, the accidents are more severe and are better for testing the various functions of the system. Finally, we define the update rates by the performance of the boards with our algorithms: ≈20 FPS with the Wandboard and ≈10 readings per second with the Arduino.

**Figure 21:**
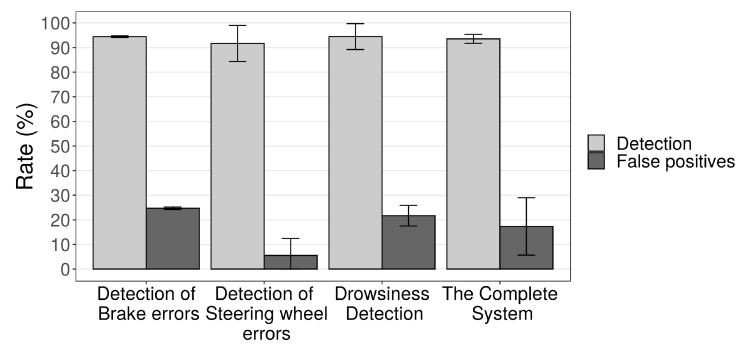
Detection rate of components and complete system.

Figure 21 shows the detection and false-positive rates of the system components and the complete system. The results present the average values with a confidence interval of 95%. The application of the EWMA graph, in the steering wheel data, guaranteed a rate of detection of sudden movements of 91.66% with only 5.55% of false positives. The brake movement results show detection of 94.44% of sudden braking at a high rate of 24.72% of false positives. This proper detection is a feature of the EWMA control graph that allows efficient and fast detection of events out of control in a process.

An observer assesses the driver fatigue, and we compared to the EWMA graphs applied to closed-eye time per reading, Figure 21 shows the graph with the results. Despite the high detection rate (94.46%), driver fatigue detection had a considerable rate of false positives, 21.68%. This false positive occurs because of the way the EWMA works. It is possible that with better refinement, the false positives decrease without a considerable decrease of the detection rate.

In addition, Figure 21 shows that the complete system has a high detection rate (93.52%) and acceptable false positive rates (17.32%), we generate these values from the average detection of the components that had the detected and false positive rates analyzed. Table 4 presents the application of a confusion matrix on test data—it demonstrates that the system’s ability to correctly predict the assessed condition is 94%, 91% and 94% for the detection of brake errors, steering wheel errors, and driver fatigue, respectively. The ability to recognize essential cases (accuracy) is 79%, 94% and 81% and the accuracy of the test which is the average of the precision and sensitivity is 86%, 92% and 87%. These results corroborate even more with the conclusions previously presented.

Once we detect some sudden movement, the system triggers an alert to the driver. Such alerts occurred in two situations: i. visual and audible alert indicating that a sudden movement occurred on the steering wheel or brake pedal, and ii. an audible alert when the driver kept his eyes closed for 1 s. The simulator tests showed that the alerts were satisfactory for results above 90% on detection in all methods.

**Figure 22:**
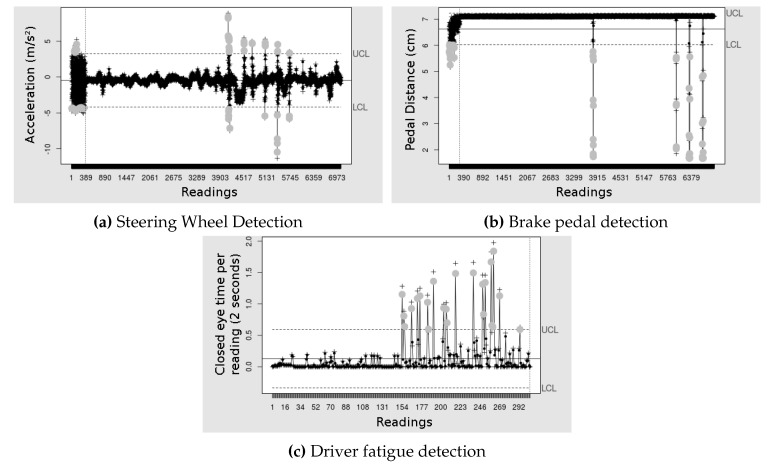
EWMA graphics for steering wheel, brake pedal and driver fatigue detection. (**a**) Detection on the steering wheel applied to the X axis of the accelerometer, (**b**) detection on the brake pedal and (**c**) driver fatigue detection from closed eye time per reading.

Finally, we present the control graphs of a test run, Figure 22. In all the graphs, very close points were considered referring to the same or false positive. Gray dots indicate out of control points, detections, or false positives. We can see in Figure 22a that all six errors were detected, three sudden movements on the steering wheel in each direction (counterclockwise and clockwise). We detect some errors more than once, due to the rapid detection of the EWMA.

Figure 22b shows the EWMA graph with the brake pedal data. The three vertical lines with gray dots to the right are the simulated errors that were detected. The control plot was able to detect the three errors, several times (near gray dots), with one false positive. This false positive was a moment where the driver had to brake but without risks.

Figure 22c shows the control chart with driver fatigue data. We can see that out of control points were detected from the middle of the graph—this is because we simulate driver fatigue from half the driving. We observe the driver fatigue moments and compare them to the graph—the gray dots show moments of driver fatigue, but they indicate some points at incorrect moments, for that reason the false positive rate was not low for this detection.

### 5.3. Qualitative Evaluation

**Figure 23:**
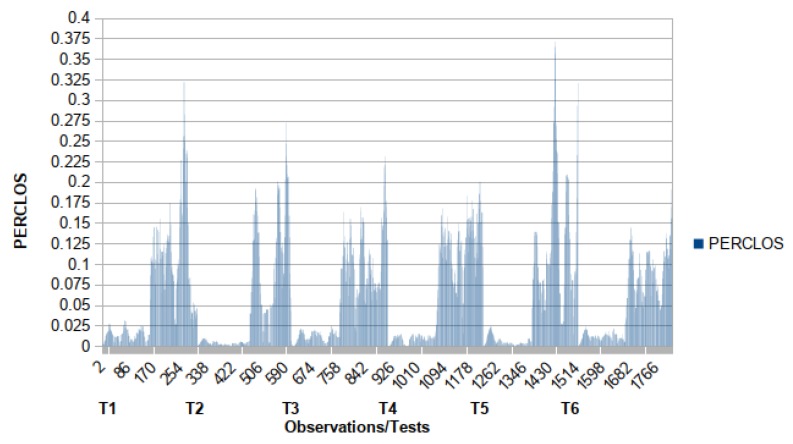
PERCLOS data with upper and lower criterion lines for three categories and single criterion for two categories.

For the qualitative evaluation, initially, we analyze the data with PERCLOS using the methodology of Wierwille et al. [24], where we have two distinct categories of wakefulness (awake and drowsy), and three distinct categories (awake, questionable and drowsy), the “questionable” category is at the center line between “awake” and “drowsy”. In Figure 23 the first ≈300 points on the abscissa correspond to observation or sample of test 1 (named as T1), the next ≈300 points correspond to samples of test 2 (named as T2), and so on. Each value is an observation, a sample, inside corresponding test. We use the same proportion values of Wierwille et al. where “questionable” is between 0.075 and 0.15 and above 0.15 is “drowsy”. The calculation performed by Kong et al. [25]:

$$PERCLOS=\frac{t}{30}\times 100\%,$$

where *t* is the duration of closed eyes.

We can observe that during the moments where the driver performed simulations of drowsiness (from half of each test) the PERCLOS considered these as “questionable” or in “drowsiness”, which evidences its effectiveness. However, the method does not point out specific circumstances where the driver had some more severe drowsiness signal, as our method provides from the EWMA.

Besides that, we perform a qualitative comparison of two works: Mehta et al. [22] and Pauly and Sankar [23] with our proposal using three analyzes: i. the types of analyses of the works and its limitations; ii. The proposal to processing the features of the driver’s face; iii. The complexity of the proposed algorithms.

The main difference between the analyses is that we tested our proposal with the use of a driving environment, a driving simulator, which makes our analysis more relevant for the detection of driver drowsiness. Besides, our detection rate was 94.46%, better than the other proposals.

**Figure 24:**
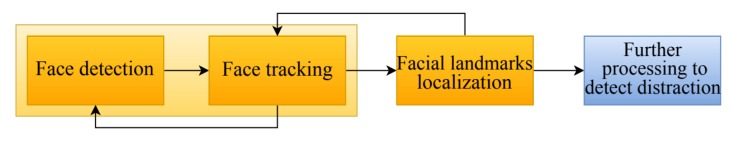
Common steps in most distraction monitoring systems according to Fernández et al. [48].

Fernández et al. [48] presents the Figure 24 and shows the typical steps in most distraction monitoring systems and the most accepted and used in the literature, such as methods based on Viola and Jones [33] to perform face detection and SVM to detect facial features, such as an eye condition. However, some additional processing is required to detect drowsiness, so this last step is significant in determining the efficiency and innovation of a method.

From this, Mehta et al. use a threshold value of EAR for the detection of sleepiness while Pauly and Sankar use the PERCLOS with a limit of 6 s, our solution is very efficient for using the EWMA to perform such analysis with results as good as PERCLOS.

A part of the computational complexity principle is the time analysis that describes the resources used computationally. For this, we can use the Big O notation to calculate the complexity of any algorithm, describing the upper bound of the increase of the running time as Rauber et al. [49] presents:

$$O\left(g\left(n\right)\right)=\left\{f\left(n\right)\;|\;\exists \;c>0,\exists \;n_{0},n_{0}\in N,\forall \;n\geq n_{0}:0\leq f\left(n\right)\leq cg\left(n\right)\right\}$$

Therefore, we compare the complexity (in terms of running time) of these monitoring vehicle drivers methods, using the Big-O notation for each of them.

*Lane departure detection*: Ahmed et al. [50] explain that the Big-O for EWMA is O(2) for each estimation value. Thus EWMA is a constant complexity algorithm. Therefore, we can say that our lane departure solutions have a constant complexity for each sample, since obtaining the data from the sensors does not depend on *N*.

*Driver fatigue and sudden movements detection*: For our sudden movements detection, we use a process similar to Lane departure detection, so its complexity is also constant. However, driver fatigue requires the driver’s eye data before performing the calculation with the EWMA. We obtain this data with the use of SVM. According to Abdiansah et al. [51], the SVM has complexity $O(n^{3})$. However, we must emphasize that almost every method of computational vision based fatigue analysis needs to obtain the data of the driver’s eye with considerable complexity, as it is presented in the related works. However, our method has low complexity after obtaining this data, making the process much less costly.

## 6. Conclusions

The work brought several contributions to the area of driving monitoring. The main one was the introduction of the use of Statistical Process Control in the development of methods to monitor driving, something not seen in the literature, thus being innovative, opening space for the development of new methods of driver analysis. From the tests performed it was possible to see that this tool can be used in the construction of methods to monitor driving data obtaining results of up to 76.92% (without constant speed) and 84.16% (speed maintained at ≈60) in lane departure detection. Our driver fatigue detection obtained results up to 94.46% and the detection of sudden movements of up to 91.66% (steering wheel) and 94.44% (brakes). The experiments performed in our scenarios presented satisfactory results, considering the average and confidence interval values of 95%. However, they cannot be generalized. We guarantee, statistically, these results since we use the same variables. Eventual other scenarios may involve new variables and, thus, consider other evaluations.

Due to the new use of EWMA from Statistical Process Control in a driving environment, this article also opens the possibility for the use of the SPC to monitor other characteristics of the driving—characteristics that were not addressed by our solutions. Finally, for developers and researchers wishing to implement solutions for driving environments, the information contained in this paper can serve as a starting point for generating new ideas and products that can meet the expectations of drivers and companies in the area of vehicles and development of solutions for monitoring and aiding driving.

For future work, we intend not only to perform lane detection, driver fatigue, and sudden movements, but also to detect pedestrians, signs, and objects on the road. We plan to combine methods such as lane detection with our method by computer vision and vehicle-based approach, verifying the conditions where systems complement each other. We can avoid accidents due to driver failures and pedestrian failures. Thus, we can carry out comprehensive monitoring of the vehicular environment. Besides, we can try to have access to a real, controlled environment to avoid the risks of testing on real highways. In order to improve the representativeness of the results, we intend to increase the number of drivers in each experiment. Finally, we intend to evaluate other non-parametric controls or Support Vector Data Description in order to render our solution robust.

## Figures and Tables

**Table 1:** Parameters used in Exponentially Weighted Moving Average (EWMA) experiments.

Parameter	Value
Factor *L* (the multiple of σ)	3
Factor of decay (λ)	0.9
Data update rate	100 ms
Vehicle speed	40 kmh

**Table 2:** Metrics from the confusion matrix on test data with Accelerometer and Gyroscope.

Metrics	Value (%)
Sensitivity	62
Accuracy	98
F1 *Score* or *F-measure*	76

**Table 3:** Metrics from the confusion matrix on the prototype data.

Metrics	Constant Speed (60 km/h)	Non-Constant Speed
Sensitivity	0.84	0.76
Accuracy	0.86	0.86
F1 *Score* or *F-measure*	0.85	0.81

**Table 4:** Metrics from the confusion matrix on the evaluated data.

Metric	Brake Pedal Detection (%)	Steering Wheel Detection (%)	Fatigue Detection (%)
Sensitivity	94	91	94
Accuracy	79	94	81
F1 *Score* or *F-measure*	86	92	87
